# Understanding key factors affecting electronic medical record implementation: a sociotechnical approach

**DOI:** 10.1186/s12913-015-0928-7

**Published:** 2015-07-17

**Authors:** Maria Cucciniello, Irvine Lapsley, Greta Nasi, Claudia Pagliari

**Affiliations:** Department of Policy Analysis and Public Management, Bocconi University, Via Roentgen, 1, 20136 Milan, Italy; IPSAR, University of Edinburgh Business School, Edinburgh, Scotland UK; Department of Policy Analysis and Public Management Bocconi University, Bocconi University, Milan, Italy; eHealth Research Group, Centre for Population Health Sciences, The Usher Institute of Population Health Sciences and Informatics, University of Edinburgh, Medical School, Teviot Place, Edinburgh, EH8 9AG UK

**Keywords:** Hospital information, ICT innovation, Actor Network Theory, Electronic Medical Record

## Abstract

**Background:**

Recent health care policies have supported the adoption of Information and Communication Technologies (ICT) but examples of failed ICT projects in this sector have highlighted the need for a greater understanding of the processes used to implement such innovations in complex organizations. This study examined the interaction of sociological and technological factors in the implementation of an Electronic Medical Record (EMR) system by a major national hospital. It aimed to obtain insights for managers planning such projects in the future and to examine the usefulness of Actor Network Theory (ANT) as a research tool in this context.

**Methods:**

Case study using documentary analysis, interviews and observations. Qualitative thematic analysis drawing on ANT.

**Results:**

Qualitative analyses revealed a complex network of interactions between organizational stakeholders and technology that helped to shape the system and influence its acceptance and adoption. The EMR clearly emerged as a central ‘actor’ within this network. The results illustrate how important it is to plan innovative and complex information systems with reference to (i) the expressed needs and involvement of different actors, starting from the initial introductory phase; (ii) promoting commitment to the system and adopting a participative approach; (iii) defining and resourcing new roles within the organization capable of supporting and sustaining the change and (iv) assessing system impacts in order to mobilize the network around a common goal.

**Conclusions:**

The paper highlights the organizational, cultural, technological, and financial considerations that should be taken into account when planning strategies for the implementation of EMR systems in hospital settings. It also demonstrates how ANT may be usefully deployed in evaluating such projects.

## Background

Throughout Europe and in other parts of the world, governments and healthcare providers are engaged in health care system reforms aimed at improving the quality and safety of care and containing costs. It is theorized that a ‘transformation’ of health care processes will come about when timely and accurate information is made available when, where and for whom it is needed [1], including the various stakeholders involved in the delivery and management of care and, in some models, the patients themselves or their family [2].

The use of effective Information and Communication Technologies and systems (ICT) underpins this vision [3] although the health care sector has been less successful in exploiting the potential of ICT than other sectors [4]. Despite the above aspirations it has been estimated that between 50 and 80 per cent of Electronic Medical Record (EMR) projects fail in the health care sector [5] outside the sphere of carefully controlled trials.

Electronic medical records systems (EMR) are designed to manage both the distribution and processing of the information required for the care delivery process, including patient care records, demographics and billing details in some systems [6]. This type of innovation is marked by a high degree of change within the organization during and after its adoption, and the speed and depth of any impact may be mitigated or enhanced by how this type of innovation is implemented [7]. Technology alone is not sufficient for ensuring the potential benefits are achieved in terms of efficiency, effectiveness, and quality of care. For these systems to be effectively implemented and their potential impacts realized, it is essential that service planners and managers understand the human and organizational processes involved in motivating change and adoption.

Recent analyses of existing research in this area [8] have concluded that context, content, and implementation process dimensions are vital to the success of technological solutions, and they have called for more research in order to understand the organizational and human factors at work in particular [9].

The research described in this paper reveals how sociological and technological factors interacted during the process of implementing an EMR system at a large teaching hospital in the UK. The paper also identifies significant issues for the implementation of such systems in the future and sets out implications for managers and practitioners with regard to the use of ICT in health care. In order to do this, the researchers deployed Actor Network Theory (ANT): an analytical tool which unpacks factors shaping the success of technologies with reference to the contexts in which they are adopted, by considering them as interacting ‘actors’ in a social network of humans and non-humans [17].

Many studies tend to see the implementation process as a “rollout” in which technology is removed from its organizational dynamics [10]. A sociotechnical approach to the study of the phenomena is necessary in the context of health care and has also been previously used to construct and analyse other processes and systems, such as quality systems [11]. It involves a variety of issues relating to people, organizational and social elements, including human-computer interaction, socio-technical, cultural and ethical concerns [12–15].

The adoption and implementation of ICT in the health care sector is a complex subject and using an actor-network perspective implies focusing on the activities of key actors as they construct and reconstruct technologies [14, 16–18], exploring what they understood about the EMR system and what “they actually do in their day to day practices to ‘make it work’ [19].

In particular, ICT innovations in organizations and in work practices within organizations can be considered to be *networks* of various related elements, such as people, tools, organizational actions and documents [14, 16].

A distinctive feature of networks is the manner in which both human and non-human actors join into a kind of seamless activity [20–22]. In this perspective, networks are associations of actors who may be human or non-human [22, 23]. Actor-networks emerge when actors identify common interests and consent to be a part of an association [24].

This process has been described as translation [17, 24]. Translation is made up of four elements (1) problematization, (2) interessement, (3) enrolment and (4) mobilization [17]. While Callon [17] described these four elements as consequential, he acknowledged that in reality they can overlap. The initial problem - in this case inadequate hospital information systems - creates space for a network. The expression of needs by key actors represents the problematization of what is required and initiates the process of network building. Interessement refers to an interest in resolving such inadequacies. The key actors who define the problematization represent a means for the imposition and stabilization of the identity of the other actors. The act of interessement may or may not lead directly to the enrolment of actors in the network. The third phase of enrolment is when a variety of actors agree on the roles and identities defined for them as actions for change. The final phase of mobilization is when the network enters into action in order to pursue its common agenda. This outcome represents a shift from dispersed actors who were not closely associated prior to the emergence of the network. This is an interactive process in which actors may negotiate, identify with others, align with others and build shared understandings. These are traces of the emergence of the network. The emergence of the network may result in adherence to particular technologies and the evolution of an information technology system with which members of the network identify [22]. A distinctive feature of the network is the manifestation of the network as a macro or super actor [25–27]. This phenomenon reveals the power of networks in action.

This paper aims to determine whether ANT is a useful way of understanding complex EMR implementation projects in terms of the various impacts on the intended outcomes, the social or procedural contingencies influencing whether or how these objectives are achieved, the reciprocal effects of IT and people on each other and on organizational processes.

## Methods

The project was conducted as part of a PhD and ethics approval was obtained from the University of Edinburgh Business School (Level 1 and 2 Research ethics applications).

No further approval was needed since the project did not require access to patients or personal data.

Members of staff interviewed (i.e. clinicians, nurses, Medical Director, CIO) gave their consent to be interviewed and for the de-identified interview data to be used for research.

### Research design

The specific focus of this study, i.e. the implementation of EMRs, is a setting where paper-based records were previously used and which can have huge consequences in terms of relations among key actors within hospitals [28]. Sociotechnical approaches favor a central role of both the actors and technologies throughout the development process [29, 30]. In actual fact, it may be that actors are only consulted a few times in meetings whose setup mitigates any real involvement of users or any real openness of the designers [31]. The approach proposed by Bergen and Berg [32] identified user-involvement as being important to foster ownership of systems that will actually match work processes. Furthermore, successfully introducing such systems into complex healthcare organizations requires a mix of good technical and organizational skills [33].

For all these reasons, a sociotechnical perspective has been adopted in this work in order to explain the mobilization of actors and the emergence of this innovation within a major hospital.

### Study setting

The hospital analyzed is a major teaching hospital in central Scotland providing acute care and surgical services to patients, primary care and community services. It offers a complete range of medical services and its medical faculty has an international reputation for cutting edge research. The hospital has 25 medical wards, including Accident & Emergency, orthopedic, maternity and specialist wards. The hospital employs 6000 staff delivering significant levels of activity to 111,000 in-patients per year, 575,000 outpatients per year and 90,000 A&E presentations per year. The scale and complexity of the hospital makes it a particularly appropriate study setting. The choice of this hospital also reflected its suitability for a wider comparative study examining the implementation of the same vendor’s EMR system at other hospitals.

### Data collection

Data was collected in several ways [34] in order to identify the multifaceted nature of situations as they enfolded and involved different actors:

- by analyzing documents produced within, by and for the hospital;
- by interviewing the key actors at the study site;
- by observing the actors within the hospital who were part of the new information system.

#### Documentary analysis

Our documentary analysis was based on official documents, reports and documents on the adoption and implementation phase in order to identify the (declared) reasons for the adoption of the system and its role. Public documents were not only examined as containers of words, images, information and instructions but also in terms of how they can influence social interaction within the organization and as a means of tracing the involvement of actors, their relationships and how their work affects and/or is affected by IT systems [35]. The documents made available for analysis were a report on the adoption of the system containing the project’s objectives and an evaluation report containing data related to the results delivered by the system.

The first document was dated December 2004 and was prepared for submission to the Scottish Government. It contains several sections, including the project’s objectives and the results and benefits offered by adopting the system. The second document made available for analysis is a report dated September 2012, containing data and information about the results produced by the system several years after it was introduced. This document provides statistics on the different types of results produced by the system over a 3-year period.

We used these as a comparator with actual observed changes. Documentary analysis was also used as a useful source for triangulation purposes since “it allows researchers to be more confident of their results” [36]. By using different data collection procedures, the researcher is able to increase the validity and robustness of results because the findings can be strengthened by cross validation achieved by using data obtained through different strategies [37].

Documentary evidence was also used to offer an insight into organizational plans and to gain an overview of activities in order to contextualize the interviews and observations [38, 39].

#### Interviews

The interview process aimed to explore the subjective accounts of people working at the organization with regard to three main phases: the selection and adoption of the EMR system, the implementation process used and the evaluation process.

Semi-structured, in-depth interviews were used as a second method of data collection in the case study setting, since these “can get close to the social actors’ meanings and interpretation, by examining their accounts of the social interactions in which they have been involved.” [40]

The interview process started with an introductory, themed interview designed to get a general idea about the hospital environment and its context in terms of activities and the types of interaction, and in order to investigate the roles of the actors (senior clinicians, senior nurses, clinicians, nursing staff, head of IT services, head of finance and control) and ascertain which human and non-human actors were involved in the EMR adoption process and were impacted by EMRs.

Several interviews were conducted with the General Director, the Medical Director and the Information System Director before starting the case study. All of these interviews were conducted in a “conversational” style [34], since they were designed to achieve an overview and a general picture both of the hospital environment and the adoption and use of EMRs.

Further interviews were conducted with a more structured approach and the interview format was divided into several parts based on findings in literature, especially to analyze the different stages in the translation process: problematization, interessement, enrolment, and mobilization [17].

“Problematization” is the identification of what constitutes a problem to be solved, as perceived by the different actors involved. The focus of ANT is the identification of ‘issues of concern’ that all actors will engage with and aim to address. The ‘obligatory passage point’ is the point of access into this collective action. As the goal is to offer a solution recognizable by others, acceptance represents an obligatory passage point for entering the network, and become indispensable in the process.

After the “interessement” (generation of interest), the translation process proceeds with “enrolment”, namely the commitment of the actors to engage in a disposition to act in light of current knowledge. “Mobilization” has to occur before this can happen.

Several studies [41] have found that there are many powerful stakeholder groups within healthcare organizations and each of these can influence the translation process and the ultimate success or failure of a system. This study took a purposive sample, helping to pick subjects on the basis of specific characteristics [34] in order to include a diversity of roles and responsibilities. We selected the key actors for the interviews based on certain specific characteristics:

- Staff profile: senior clinicians, senior nurses, clinicians, nurses, managers, CIO, in house IT staff (clinicians, nurses, General Director, Medical Director, CIO)

- The number of years working at the organization: before the adoption of the EMR system at the very least, after the adoption of the EMR system. For this reason, many senior staff were included in the sample even if junior clinicians and nurses were included in the observation process.

Some interviewees were specifically selected for their ability “to shed light on a particular aspect of the behavior under investigation” [42]. These included the General Director and the Director of eHealth. Other interviewees were selected using the snowball technique: the initial respondents were used as informants to identify others with previously defined characteristics: staff profile and number of years working for the organization (since the adoption of the EMR system).

The Director of eHealth was identified as playing an especially relevant role in the overall process from both a technical and strategic point of view.

Clinicians and nurses were interviewed from the 4 departments included in the study. The four departments were selected because each of them is representative of a specific “area” inside the hospital. From an organizational point of view, the hospital is divided into 4 main areas that have adopted Electronic Medical Records. As a result this study sample includes:

- Medical Area: all wards providing general health care;
- Emergency Area: wards providing emergency care;
- Specialist Medicine Area: wards delivering specialist health care, mainly on an acute admission basis for inpatients or day patients;
- Maternal and Infant Area: wards integrating different aspects of patient care (such as the maternity ward and pediatrics).

We selected one ward from each area. Each ward can be considered as representing the overall area in terms of beds, patients and number of employees (Table 1).

Two of the areas selected were included in the study sample because of their specific characteristics: A&E was the first ward to adopt the EMR system and the maternity ward was the last to do so.

Some pilot interviews were carried out. These were also recorded to make it possible to reflect on them at a later date and to identify the processes at work in the implementation. This also helped to prepare the interviewer by trying to anticipate any ethical issues and reduce any influence the interviewer may exert during the interview process.

**Fig. 1:**
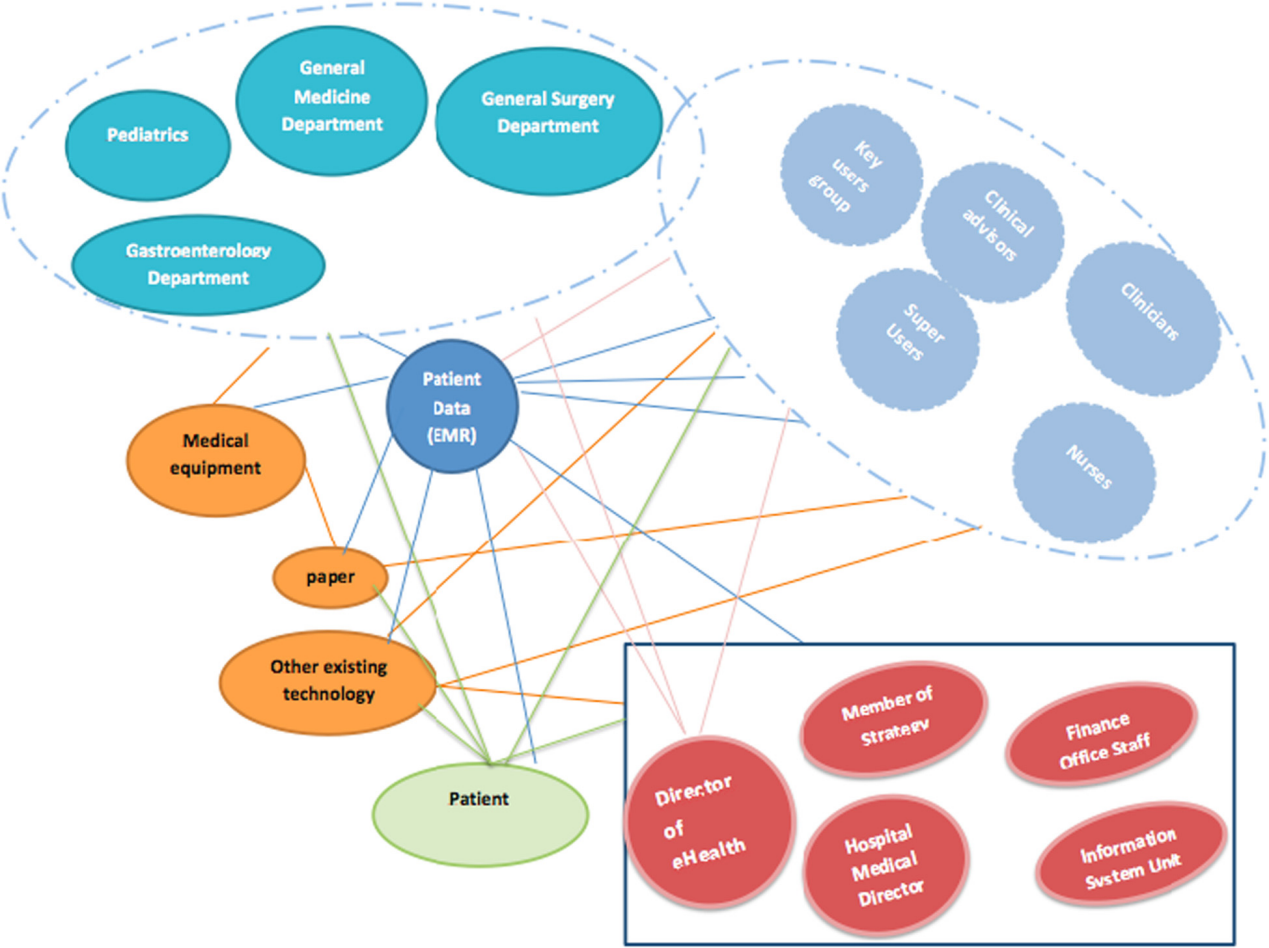
Actors and networks in relation to the introduction of the Electronic Medical Record

Figure 1 illustrates the different actors and networks involved in the analysis, highlighting human actors (in blue and light blue), organizational actors (in red), and technological actors (in orange).

In order to analyze the interviews, we recorded all meetings with the stakeholders on audio tape. Each interview took an average of 45 min, with some lasting over an hour and the shortest lasting 40 min. Each interview was then transcribed and the data was analyzed. After familiarization, recurring themes and ideas were identified and a coding index was then developed with themes subsequently sorted into broader categories and key themes. In a first phase, the transcripts of interviews were manually analyzed: this step was necessary to familiarize the researcher with the text and start to develop lists of codes for analysis. The analysis primarily coded the types of response of the respondents in order to highlight any common concepts proceeding in an inductive way. Subsequently, the codes generated were analyzed in light of a more detailed analysis of literature and theory, and brought back into macro-categories according to a concept-driven approach and to the main findings of the literature review [43] (Table 2).

The analysis was inductive. In this case, themes (or nodes in NVivo terminology) were identified in the transcripts regardless of their occurrence in the interview guide, very much influenced by an ANT approach.

Furthermore, although it has been argued that using specialized software is not necessary with a small dataset because a word processor is sufficient [44], NVIVO 8 software was used in this study to have enhanced access to data and achieve greater transparency and consistency.

#### Observation

Observations were noted during the research team’s visits to the hospital with regard to the actions, reactions and interactions of actors within the hospital, and specifically in relation to the EMR system. There was limited time for direct observation of the EMR in action within this study and any such observation is non-participant and “very much on the periphery of interaction” [45]. As such, the use of observation is basically supplementary, with the purpose of augmenting data from interviews and documentary materials. It may include observation of work, of everyday life in the organization before and after interviews, or during breaks (such as coffee breaks and meal times). The observation process started from general observation of the hospital environment. It entailed observing interaction between clinicians, nurses and other staff, and between these and the patients. This facilitated the observations of different situations in several departments since modern organization “takes place in multiple fragmented contexts” [26].

Observation as a method of inquiring is a valuable means of studying relationships among people, facts and the organizational context – both at the micro and macro level [46]. In this study, we focused on the ANT premise that humans and non-humans are jointly involved in the making of the social world. The observations were overt to the medical, nursing and administrative staff and covert to the patient. In particular, the interview phase offered an opportunity to develop the necessary rapport with interviewees and so facilitate both access and the observation process.

Multiple types of observation, including object-/activity-/person-oriented [15] were used to complement interviews, also with the purpose of triangulation and to contribute to a deeper understanding of the sociotechnical processes involved in adopting the new technology.

The researcher used “shadowing” as a method of observation and information gathering, moving from one point in a context and network to another with the shadowed person. This helped to focus observational data collection activities around the software and associated human actions (e.g. direct software use or progress meetings).

In carrying out the observations, the researcher noted settings, actors, activities and impressions relating to the observation. Spending time on the ward, especially in reception, offered a unique opportunity for observation. The researcher was fully immersed in the context and could observe staff and patient behavior in relation to the EMR system (as reception is a major point of interaction on the ward).

Clinicians and nurses were generally approached while they were working and the researcher had the unique opportunity to observe how they use the system and note if they appear to be confident with it; this helped to gain a better understanding and clearer vision of the context and so understand what people say during the interviews. Participants in the study were also asked “to recall critical incidents or examples of system use” as suggested by Kaplan in her work on the evaluation of medical systems [12].

## Results

We collected data through multiple methods, documentary analysis, interviews and observations, in line with Latour’s [47] guidelines that recommend an immersion in study settings in which everything is data and the careful noting of all phenomena of interest within the study setting.

Interview data was obtained from a total of 19 different participants, two official hospital documents were identified by the key respondents and subjected to documentary analysis. The research team also carried out 30 h of observations, which were recorded in written field notes.

A summary of the data collected is provided in Table 3.

**Table 1:** The study sample

Area within the hospital	Ward
Specialist Medicine	Gastroenterology Ward
Medical	General Medicine Ward
Maternal Infant	Maternity Ward
Emergency	Accident & Emergency (A&E) Ward

**Table 2:** The interview sample

Role of participants	Key	Number
Member of Strategy Board	STB	1
Director of eHealth	HD	1
Finance office staff	FO	1
Clinical advisors	CA	4
Senior nurses	HN	4
Senior clinicians	HC	4
Receptionists	R	4

**Table 3:** Summary of data collected

Documentary analysis	Interviews	Observation
• Official documents, reports, and documents on the adoption and implementation phase were considered;	• Each type of staff profile was considered;	• Observation during the research team’s visit to the hospital;
	• 19 people interviewed;	• Object: reactions and interaction of actors, specifically with respect to the EMR system;
• Main documents: (1) report on the adoption of the system containing the project’s objectives; (2) evaluation report containing the results of the system.	• Each interview took an average of 45 minutes;	• 30 hours of observation.
	• 35 pages of interview transcriptions.	• 20 pages of research field notes;

### Analytic approach

Data from the interviews and observations was first coded descriptively according to high level themes such as stakeholders, settings, stages of implementation, drawing on the results of a literature review. Next, it was analyzed with reference to the issues and stages described in ANT theory, as well as related concepts arising from our literature review, and organized into macro-categories [43]. For example, this helped to describe the building of a network around the EMR as a non-human actor by exploring how the articulation of the problematization of poor hospital information systems led to rapid identification with the technology (interessement) and into the overlapping third and fourth phases of enrolment and mobilization. The following sections organize qualitative results firstly with reference to these stages, and then focusing more on the last stage. The main impacts produced by the EMR system are analyzed from the more general perspectives of the technology, the organization and the particular stakeholders involved.

### Problematization: weak hospital information systems

The hospital suffered from the lack of integrated information due to the different systems in use at the hospital and the lack of data exchange between them. The situation was marked by the resulting issues: first, there was limited functionality when ordering test results, as the process was still paper-based. Three separate radiology systems existed that did not support the integration of PAC and there was no single Master Patient Index for covering these services. Furthermore, eight different patient databases were in use with different patient numbering systems. This meant that patient information was entered into multiple systems for each patient episode and could not be linked or shared electronically.

The hospital had three separate patient administration systems, with the majority of the patient’s clinical letters written in MS WORD and therefore not linked to any electronic patient record, and two unrelated A&E systems that were not integrated with the rest of the hospital. For this reason, a great deal of important clinical information was only available to A&E and not to other parts of the hospital, except in the form of paper case-notes.

Furthermore, the basic results reporting facility available to clinicians was hampered by huge clinical weaknesses that reduced its usefulness and capacity to support clinical governance, and the hospital was unable to provide a patient’s clinical letters (e.g. discharge letters) and radiology reports generated at regional level.

The Director of eHealth described the situation prior to the adoption of the EMR project very clearly:

*“Up to 2004 the situation at [the] hospital was marked by inefficient processes; clinical decision making was based on a minimal information service that was not patient friendly and bore an unacceptable level of risk.” (Interview with the Director of eHealth).*

He also added:

“*To give you an idea, 5 years ago we had 3 A&E systems, 4 radiology systems and 3 separate patient administration systems. I think we had a total of 17 systems doing almost the same thing and patient details were registered in different systems and it was impossible to get the full picture”. (Interview with the Director of eHealth).*

Another interviewee offered an interesting opinion, mentioning the need for patient information in real time throughout the entire hospital.

*“The main problem was that paper records were not reliable. We didn’t have any choice; we just needed a better system. At the time, we had a huge storage problem with storing these records. We can’t store them on site, they have to be somewhere else, and then we’ve got to wait until somebody finds them and we periodically lose them. So having an electronic system means that we’ll get easy access to the patient’s record, when the patient is there. Instead of having no records and information about a patient because they’re in a storage facility.” (Interview with a clinical advisor)*

These interviews describe the first moment of translation, when the identification of what constitutes a problem to be solved occurs.

When asked interviewees to identify the person who was most influential in encouraging EMR adoption and who played the most relevant role at this stage of translation leading to the enrolment of other actors in the network. All the interviewees, including the members of the Strategy Board and the advisors involved in the project, mentioned the same person: the Manager of the eHealth Programme, who was the EMR project manager at the time of the adoption. All of the interviewees, including clinical and nursing staff who did not know him personally, mentioned him as the leading actor within the project and said he encouraged EMR adoption, playing a major role in making it happen.

He represents the ‘focal actor’ capable of defining the identities and interests of other actors, and of establishing itself as an obligatory passage point thus "rendering itself indispensable" [17].

### Interessement: the EMR solution seen as non-human super actor

Interessement represents the second moment of translation, which involves a process of convincing other actors to accept the definition of the focal actor [17]. The different actors we interviewed suggested various reasons for the adoption of the system. As noted earlier, we approached the EMR application in health care organizations from the perspective that it can never be a process of simply installing and using a new technology [48] and that different groups within an organization may see the same technology as achieving different goals.

The interviewees at the hospital identified three main reasons for adopting the system: the need for information within the organization, to integrate information from different systems, and to improve value for end users and patients.

The main push for adoption came from the clinical staff, not management staff, and the preferred system was chosen from two potential solutions during a workshop attended by clinical staff. This emphasizes that the adoption of the system was marked by “consensus” regarding the need to introduce the system and the system selected [49].

As a result, we investigated how the selection of the system happened. It appeared to be based on a “user-centered approach” during the selection and adoption phase, listing the needs of end users, involving them early on, thoroughly and systematically [50] [51]. A participatory process in selecting the EMR system represents a distinctive way to generate and improve commitment to the project within the organization. Conversely, if this process is imposed, it can generate user frustration and have a negative impact on the implementation process and on the overall use of the system.

We questioned several of the people in our sample, but a clinician from the A&E ward offered the most instructive answer.

*“Before choosing the system we adopted, we had the opportunity to look at several options” (clinician from the A&E ward)*

One option was to identify a new software vendor; the other was to use a company that had already supplied a number of core systems. The name of the other potential supplier was not revealed, for reasons of privacy and ethical considerations.

The clinicians unanimously decided to adopt the first option. The 60 people who took part in the workshop were invited because they were involved in the process in various ways and this was the final part of that process. Thus, the system selection and further developments were user-centered, based on user needs and on a participative approach.

In fact, in order to select the system, the hospital proceeded in a clear way:

*“We undertook the usual evaluation: supplier presentation, discussion, cost - benefits analysis. We had to produce a case study for submission to the Scottish Government. The preferred option was chosen halfway through a workshop we organized and I think we had about 60 people at that workshop. The majority were clinicians and all the clinical staff put their hand up for the new system.” (Interview with the Director of eHealth)*

Some interviewees identified other reasons for adoption, emphasizing the relevance of the system and clarifying the aspects leading to system adoption.

According to a member of the Strategy Board:

*“The overall project is the reflection of the commitment and dedication of both the hospital and supplier teams, working together to make this happen. As part of the move from the hospital’s current systems, over a million patient records were transferred to the new system from both the previous patient administration and A&E systems. Previously these systems were operated independently from each other and led to unnecessary repetition of data entry during the patient care process. The new system will allow all or part of the patient records to be available to clinicians at a time and place when it is needed, supporting high-quality patient care through faster access to patient information.” (Interview with a member of the Strategy Board)*

According to a clinical advisor working for the eHealth department and in charge of supporting and supervising the maternity unit:

*“From an organizational point of view, clinical staff wanted to have more information about their patients, let’s say about mums, such as more strategic info coming out from the system, so they could do more work with planning activities also for long periods” (Interview with a clinical advisor working for the eHealth department).*

The new EMR system is a “connected healthcare information system” with EMRs integrating clinical and administrative modules. As emphasized by the interviewees, the system offered the opportunity for *adequate administrative information for care and operational managers*, since it helps to streamline the collection and the processing of administrative data with minimal workload for health care professionals.

*“The new system lets you collect and store huge amounts of data. It supports administrative processes related to patients’ data, assuring information is broadly available, timely, reliable and always correct, or as we used to say: ‘correct first time’”(Interview with a member of the Strategy Board)*

When asked to identify the main reasons for adoption, a senior clinician from the General Medicine ward explained that:

*“This hospital was designed so that the laboratory service has a single central booking area with few staff…so it was looking for help to manage this and electronic ordering provides a way of doing this …It was the possibility to get all the information and demographic data so they do not have to enter this data and look for it in others papers.”(Interview with a senior clinician, General Medicine ward)*

This enables multidimensional integration that is particularly relevant for the implementation of EMR systems, enabling the full scope of the clinical and administrative information relating to a patient to be collected together. In this way, the EMR provides a patient-centric display of all available information.

The interface of the system made it simpler to use and to apply. It was clear that this EMR system would be a much better solution in terms of integration compared to the product offered by the other supplier. The Director of eHealth added:

*“I think they felt that the first supplier was more able to list what the organization needed, while the other supplier was very rigid and said what they should do and what they should not, so they had very clear reasons for choosing this EMR system.” (Director of eHealth).*

### Enrolment and mobilization

In this study, the success of both the problematization and interessement phases, which illustrate the relevance of a participative approach in the decision making process, had a significant influence on the enrolment and mobilization phases, which went hand in hand in this study. The manner in which enrolment and mobilization progressed is described below through the experiences of different actors.

In the enrolment phase, a variety of actors agree on the roles and identities defined for them as actions for change: new roles were defined to lead to system implementation with the hospital.

The implementation of the system was structured: it started by implementing the most relevant functions across the entire hospital, and then continued by piloting additional functions in single wards in order to test them and to get feedback from staff working on the selected wards. This helped make any adjustments based on results and the progress made when using the system. Furthermore, by selecting wards for the pilot test of the new functions, they could analyze how the system worked in different scenarios: inpatients, outpatients, emergency ward. The clinical advisor on the General Medicine ward said:

*“An area we are currently starting to pilot is the online review of results….Currently almost all results return electronically to EMR system from the laboratory What we are looking at is how we can read these results and we are going to test how we think this can work for us.*

*We are piloting this function in an inpatient ward, an outpatient ward and the Emergency department. We would like to understand how well it works in the different scenarios: inpatient, outpatient, emergency. Accident and Emergency is a ward where the system is put to good clinical use.” (Interview with the clinical advisor on the General Medicine ward*).

The system implementation was managed by the “Information System implementation team” and was overseen by a “Program Board”, namely a group that initially met once a month and still meets on a monthly basis to oversee the implementation, formulate advice, verify if any help is needed and provide it in this case.

Other key roles were identified during the implementation process and people were appointed to these new positions. Clinical advisors were identified who could assist with the implementation of the system in a specific ward. They worked for a specific ward but held different positions at the time of the adoption. For example, we interviewed the clinical advisor for the maternity ward, who explained how this change took place:

*“Well when…when I came into the project, they were implementing electronic medical records in the maternity unit. And I was employed at the time as a qualified midwife on the maternity unit, ward 209. Then in July 2008 a job was advertised for a clinical advisor to come and join the project”. (Interview with a clinical advisor working for the eHealth department and in charge of supporting and supervising the maternity unit).*

This suggests that a job advertisement was posted to support the implementation of the system within the unit. No external people were taken on by the hospital to support the implementation process; instead, people were selected who already knew the services provided by the hospital and by these particular wards and were moved to cover the new positions.

Clinical advisors perform specific activities:

- They accurately represent and apply the best practices and methods of clinical and technical expertise and clinical and technical leadership of the project by conceptualizing, developing and administering training and service delivery to improve strategies, projects and tools;
- They evaluate interventions by developing, enhancing or reinforcing the use of new systems to build the capacity of staff, consultants in technical content areas and provide training and service delivery;
- They supply technical input on strategic program and system planning, design, implementation and evaluation.

Furthermore, ‘implementation staff’ were defined to carry out the new system implementation. This support team was initially quite informal. According to the clinical advisor in charge of coordinating the implementation staff, the role of the new team, consisting of 3 people, subsequently grew and is now:

*“Picking up on the mistakes that people were making….In fact, looking at the EMR system implemented, we realized we needed to go back and support staff, we had to show them what they were doing wrong and correct it. So that’s how we came about. I have been appointed to this new position since January 2007, before that I worked as a nurse in the surgical unit. My contract was due to expire in 2009 but then they asked me to continue and help get staff on the wards using the EMR system properly”. (Interview with the clinical advisor in charge of coordinating the implementation staff).*

After extensive training courses arranged at the initial phase to guide the introduction of the new system, support staff managed the training delivered on the wards. A “key users group” was also identified, namely about 200 people from across the hospital who were particularly interested in the EMR system and its strategic development, and “super users” were appointed on each ward. These are clinicians or nurses who are capable of training other people; they work on the ward and are very motivated so they act as “local facilitators” in each department, supporting staff and training new staff.

The implementation group set up a skills-based system in collaboration with the eHealth department, to train the super users on training techniques. The implementation staff also checked their knowledge of the new system and issued a super user certificate so they can operate in a specific ward.

*“We have it all down on paper, with check boxes, until it’s electronic and then they’ve got jobs they can do with their staff and their departments, to make sure they’re competent.” (Interview with a clinical advisor).*

Super users were very interested in the new system; they were often already conversant in ICT so their experience was a sort of “knowledge tool”, meaning they had developed good skills in using the system in the past by attending training programs held by the implementation staff. They offered themselves as volunteer “super users” for their ward. This implies that people within the department were no longer required to attend training courses outside the ward. As a result, they did not have to leave their place of work and could get all the help and training where and when they needed it. We should remember that people choose to become super users but do not get any extra money for doing this work.

It sometimes happens that the unit managers identify who could be a super user, however according to the chief of the “implementation staff:

*“Ideally, we’d like people to volunteer to do it…” (Interview with the chief of the “implementation staff”)*

Being a volunteer not only means they offer themselves spontaneously, it also means they will not get any financial reward. It is a way to certify their skills in using an EMR system and could be helpful for them to add this information on their curriculum vitae when applying for another job.

This can also be recognized as a key strategic role supporting the implementation phase. Previous studies (such as [52]) have also found that knowledge about a specific health information system is best communicated by persons who are familiar with clinical applications and functions and who are able to integrate the ways of performing tasks with the daily working praxis.

This method of training focused on learning how to use the system by looking at the existing work practices within each ward. The introduction of such systems produced effects on existing work conditions and users needed to learn how to integrate electronic and interpersonal communication of information.

Users needed to feel that the value gained from the adoption of the new system will be higher than the challenges and the effort spent learning how to use it and for changing the previous way things were done [53].

In this specific stage of translation, the role of super users within the departments was extremely precious but also challenging. All of the nurses we interviewed defined the training activities done by super users on the wards to be very helpful and acknowledged that they had more problems and wasted more time trying to figure out how to manage some of the system functions before this role was introduced.

The senior nurse, who is a super user for one of the wards in the analysis, stated:

*“I started using the EMR system five years ago and I found it very simple and intuitive to use…I had some experience in using a PC and maybe this helped me. Then, before the system roll out within our department we attended some training courses; let’s say 3 full days training. Then, we started using the system and last autumn the clinical advisor delegated to our wards from the eHealth office asked for people interested in attending a course for training other people in using the system. I usually help my colleagues and so I offered as a volunteer and my colleagues also suggested my name. I think the EMR system is a very useful tool and I think it has changed not only how they manage patient records but also how they communicate with each other, the way we provide patient care services, and perform job responsibilities. For these reasons, I decided to also take part in the skills assessment test, since I think the system can effectively change the way we work and help our patients”.*

However, not all the people reacted in such an enthusiastic way as super users.

According to eHealth department advisors, and based on what staff said themselves during the interviews, many people were skeptical because they were asked to do something that they didn’t do before.

*“It’s like any change, people automatically say, oh… They’ve got a fear of change. For the majority of them, when they realized all they had to do was a few clicks on a screen, then, most of them thought well, is that it? Okay, we can do that. Another set of people was more unsympathetic to using the system and it took us a long time to convince some of them (that) what they were doing, the way they were working with paper records was actually taking longer and if they would just click on the screen, that’s a lot quicker!! For example if the nursing staff have to do blood exams on patients, the doctors would write the forms out. So they have to wait for the forms, and then they would go and take the blood. Now they do it all online, and there’s no forms, no paper involved. A little label prints out, with the patient’s details, they stick it on the blood sample, and away it goes, that’s it. If you enter the ward and say to them, right, we’re gonna take all that away, and you’re going back to the old system, and you’re gonna do it on paper forms, oh no, no; no, no, don’t do it; don’t do it. They thank me afterwards. So although they complain that it was time consuming initially, they don’t want to take it away either.” (Interview with a clinical advisor from the gastroenterology ward)*

The way in which people reacted to the adoption and implementation of the system was also influenced by their age and attitude to ICT in general.

When the implementation started, many people did not use IT. Younger staff knew how to use a PC but older doctors and nurses were a bit more reluctant. After some time, they started to recognize that it can help to cut their workload since a lot of information is stored on the EMR system: clinical letters for nurses, patient discharge letters for doctors, test results.

Doubts were initially raised on the wards about safeguarding patient privacy when using the EMR system. In some departments, staff felt that the initial training was poor:

*“We were greatly criticized for training, they felt training was, was very poor.” (Interview with the Director of eHealth)*

But the eHealth department and the strategy board understood these needs and came up with solutions, such as implementation staff and the role of super users on the wards.

User-involvement in this case represents an important element in the enrolment and mobilization stage: it helped to promote enrolment and foster ownership of the system. It is not enough to include a few potential users in the project group to have them negotiate the system specifications and discuss implementation plans and the achievement of change in a meeting: it is necessary to include people at different levels, also defining specific roles and the activities to be performed. As the interviews brought to light, people within the wards started to offer themselves as volunteers to be involved at different level and covering different roles in the mobilization of the new system within the hospital.

Focusing on the mobilization phase, namely when the network starts to speak as one and to produce some effects in the hospital, we attempted to achieve an in-depth understanding of the role of the system within the organization and how it affects the conditions at work.

Taking the impacts produced by the mobilization of the network, four main themes emerged from our analysis:

- The health care delivery process;
- People working within the organization;
- Patients
- Relationships with institutional and other stakeholders

We discuss our findings on the impacts in more detail below.

Based on the interviews carried out, staff working on the wards (clinicians, nurses, receptionists) had the most informed position for answering the questions related to the evaluation and these are illustrated by selected quotes based on significance, in terms of the relevance for the different actors and for the overall process, and frequency, namely how often they occurred.

#### The health care delivery process

One of the most highly acknowledged benefits since the adoption of the EMR system at the hospital is the time saved as a result of the faster sourcing of information and data related to previous admissions via the EMR system. Most of the clinicians we interviewed maintained that the adoption of EMRs reduces the waiting time for laboratory test results and enables diagnostic images to be viewed immediately, saving time during the execution of daily activities.

*“Compared to the initial phase, the system is now used with more functions, such as for example blood tests, X-rays, and so on. At the start, it was only used for a few types of information and later they started to use it for more functions. It was helpful for some activities and it also helped to save time…it is much easier working on the ward.” (Interview with a head clinician of the general medicine ward).*

The adoption of EMRs produces important results in terms of the accuracy, completeness, ease of understanding and reliability of information. At the time of data collection, the respondents had some experience of using the system and started to be aware of some adjustments compared to the initial phase.

A clinician, talking about his own evaluation of EMR, gave an interesting answer. He sums up his own usage of the system as follows:

….“*first of all, I think that the adoption of the system has improved the accuracy and completeness of data. This means that I can have access to more complete information in terms of laboratory test results, X-rays, and I get them more rapidly compared to before the adoption… I can display them at any time and at any place…… Furthermore, it has helped save time when searching, editing and storing documents. ….The system enabled fewer documents to be printed. It also reduced the need for further exams and investigation… “(Interview with a clinician, A&E ward)*

According to the majority of clinicians interviewed, the system helped them to (i) check results; (ii) provide alerts about allergies; (iii) identify the location of patients on the ward; (iv) send letters to GPs.

In particular, one clinician said:

*“It helped in ordering investigations, and in knowing which patient is in the department, where he is and who he has been referred to. I think it “improved the patient flow”, in terms of how the process happens when a patient comes to the front door and it is much smoother and easier to manage with the EMR system. After visiting a patient, I simply type into the system what I have written on a sheet for the GP and if I have to prescribe something, I just prescribe it.” (Interview with a senior clinician, gastroenterology ward)*

The interviews with the nurses in the study sample revealed that the adoption of the system also helped by producing more legible notes that are easy to understand without the need to deduce or decode clinicians’ handwriting.

*“Now, it is easier to understand what clinicians write, without the need to interpret their handwriting. This makes me feel more comfortable when doing my job.”(Interview with a senior nurse, A&E ward)*

Results in terms of impacts on risk management are mainly linked to the presence of alerts, whereas the interviewees refer to the reduction of errors associated with the integration of information between different wards and throughout the hospital in all phases of patient workflow.

*“… more evident benefits were revealed in terms of error reduction. The system shows previously recorded allergies and alerts and helps in recording any newly identified cases” (Interview with a senior clinician, gastroenterology ward)*

Comprehensive medical information not only provides the healthcare provider with alerts, but also with information for avoiding unnecessary invasive clinical tests.

Clinicians reported feeling more confident with the information they receive before they decide on a clinical diagnosis: they have safer and more reliable information thanks to the EMR system. Interestingly enough, we found that respondents acknowledged significant improvements at work in terms of the improved ability to plan admissions, more accurate diagnosis and treatment and the reduction of errors in prescribing tests and compiling reports.

*“We have a number of things that follow from the drivers. The integration with PACS has really been one of the major successes, we really had good results and clinicians were very happy about it …since we started to provide laboratory results, final reports and images all together…they were saying that they need to…they need to have information at their finger tips. From the administrative point of view, we have electronic referral receipts from the GPs…Before that, to find a referral, the administrative staff had to go to the referral system, print off the referral from the EMR system, type the name, enter the details into the system to make them available. So it was a very time consuming process.” (Interview with the Director of eHealth)*

Detailed sub-themes and the related interview transcripts are summarized in Table 4.

**Table 4:** Sub-themes and the related interview transcriptsᅟ

The health care delivery process:
– Time savings in undertaking activities, such as searching, editing and storing documentation or concerning the waiting time for laboratory test results
– Information quality in terms of the accuracy and completeness of data
– Improvement of diagnostic and therapeutic activities
– Accessibility, since the system allows for the checking of images or reposts at any place and at any time
– Error reduction, since the system shows previously recorded allergies and alerts and helps in recording any newly identified cases
– Cost savings: the system is paperless and it cuts the need for further exams and investigation

Some interviewees also acknowledged the importance of the system for improving information sharing and the integration of data among different hospital sites, suggesting positive ‘whole system’ effects.

*“The biggest impact now is that we can get information in real time, and that was not something we ever had before. There are a lot of improvement programmers that have been going on since the initial adoption. The EMR system has allowed us to be able to look at the patient pathways and measure them all the way along… and measure the time people are waiting. And then the other big opportunity offered by the system is the information sharing and integration of data among the different hospital sites…we have so many hospitals” (Interview with a senior clinician, general medicine ward)*

This led to organizational changes that currently allow for better planning of admissions, more accurate treatment and fewer errors in prescribing tests and compiling reports.

#### People working within the organization

The interviewees on the different wards agreed that the most significant effects on people working within the organization were at *“*communication level*”,* in the form of improved interaction between clinicians and nurses on the same ward, and between different units and hospital sites.

*“I think the system is a very useful tool and I think it has changed not only how they manage patient records but also how they communicate with each other, the way we provide patient care services, and perform job responsibilities. For these reasons, I decided to also take part in the skills assessment test, since I think the EMR system can effectively change the way we work and help our patients” (Interview with a senior nurse, maternity ward)*

The eHealth Director offered a very clear answer to this question:

*“Of course, the adoption of the system affected people working within the hospital and their daily activities. When we started the project, many people did not use IT. A doctor does not use IT for his job…there is no reason to use a PC.*

*For junior staff, they know how to use a PC but older staff, like doctors and nurses, were a bit more reluctant and we continue to have that, even if we reduced their workload since a lot of information is stored on the EMR system….” (Interview with the Director of eHealth)*

Furthermore, both nurses and clinicians recognized that the adoption of the EMR system helped them to get better and more complete information, including information on previous patient admissions, and supported interaction and communication between members of staff, helping to link the different actors in the network, as discussed below.

*“It has definitely improved relationships between clinicians and nurses…. in the sense that we can all access the same information without going around and asking for details, or results and information in general terms.” (Interview with a senior nurse, general medicine ward)*

*“We can also check and get all the information about previous attendances, and about particular problems we need to be aware of, such as if children are on the protection registry or if they suffer chronic problems, such as diabetes, or if they have any allergies.” (Interview with a nurse, A&E ward)*

There was general consensus that the adoption of the system did not enhance the commitment of clinicians and nurses.

*“The system did not affect clinicians’ and nurses’ commitment as this is not related to the use of the system.”(Interview with a senior clinician, general medicine ward)*

Staff involvement and their level of commitment seems to be independent of the adoption of the EMR system. This may or may not be present within an organization, but is not related to the adoption and use of EMR.

#### Patients

When asked about the impacts that the system produced on patients, there was general agreement that patients are not aware of the use of the system. In the maternity ward we studied, clinicians and nurses agreed that patients know the system is in use and this made them feel safer. The ward staff we interviewed reported that patients staying on their ward tend to be younger than most people admitted to hospital; as a result they are more familiar with computers and seem to expect care to be computerized.

*In Maternity, we are dealing with a generation, because obviously it’s young reproductive women that are having babies, so you know, when I started midwifery, over 20 years ago, the people I was looking after were the same age as me…Whereas now, the people that are coming in to have babies are 20 years younger than me. And they’re a generation who have been brought up with computers. They use computers at school, they work with computers in their job, they might be IT people, they are involved with computers. So they seem to expect care to be computerized. They don’t expect you to be sitting writing lots of sheets of paper. So I think their acceptance of an electronic system is probably much better than in the past. So from that point of view, I think they are accepting it, and it doesn’t matter.” (Interview with a senior clinician, maternity ward)*

However, many of the clinicians and nurses we interviewed were concerned about the possibility that confidential patient data could be disclosed and used improperly. In particular, a clinician on the gastroenterology ward said:

*“I’m especially worried about it being possible to access patient data from outside the hospital, when working from home. This may help by improving the allocation of a clinician’s time but could lead to privacy issues: laptops can be stolen and confidential data could then be accessed by unauthorized persons.” (Interview with a clinician, gastroenterology ward).*

Given the bearing of the matter, privacy issues linked to the adoption of EMRs were a concern for certain members of the network. Such tensions are typical of the unsettling process of network identification and network building [17].

Based on the analysis carried out, it is possible to classify the privacy reservations associated with EMR systems into 3 macro-categories: i) apprehension about *inappropriate delivery of information,* due to unauthorized users accessing data and using it with purposes that conflict with organizational policy or unauthorized database access by people from outside; ii) concerns about the information exchanged between health care organizations and other institutions, such as primary care organizations, governmental organizations, or pharmaceutical industries; iii) concerns about the possibilities of data confidentiality loss.

Indeed, the clinicians and nurses we interviewed were concerned about the possibility that confidential patient data could be disclosed and used inappropriately for a variety of purposes, and worried that it is possible to access patient data from outside the hospital, when working from home. Attempts have been made to resolve these concerns about privacy, confidentiality and security issues related to EMR use within healthcare organizations in terms of regulations, standard and guidelines, code of conducts, codes of ethics, technical solutions. However, this major issue has not yet been resolved, and permanent solutions are needed that take all of the previous issues into consideration in order to develop a more effective approach to ethical issues for all the situations.

#### Relationships with other stakeholders

When asked about this impact dimension, six interviewees reported that the system had the potential to strongly affect relationships between professional and institutional stakeholders through better information and data exchange, even if this is only noticeable in the long term [54]. All added that these influences would be mediated by the fact that NHS Scotland had signed a contract in 2012 with the vendor of the hospital system, to develop a new national patient management system, as the eHealth Director stated:

“*In this country, in a number of years, when the system will be implemented at all other sites, the system will help to share the same view …I think that in terms of full EMR, we have to wait a few years after the hospitals go together, but I think that it can happen. Clinicians and patients will both be winners from a system which will track patient journeys from referral to discharge. It means clinicians will have easier and quicker access to medical records and patients will benefit from having more time with healthcare professionals.” (Interview with the Director of eHealth).*

Based on the official documents collected and analyzed, a consortium of five Health Boards in Scotland created a team of more than 160 users to agree on requirements, and selected this system as the national patient management system in a rigorous two-year procurement process.

An official document reported that when the contract was signed, the Chairman of the eHealth Program Board of the National Health Service in Scotland said: “We believe that the system will play an important role in streamlining patient services leading to faster diagnosis and treatment while enhancing patient safety.”

The new system will help to accelerate and improve the effectiveness of patient care throughout the country by ensuring patient information only has to be entered once for it to be immediately accessible by authorized staff in other health care settings. The new patient management system includes administration of hospital and mental health patients, confirmation of orders, results reporting and clinical support tools. A number of optional modules are available for accident and emergency, electronic prescriptions and administration of drugs, pharmacy management, maternity and neonatal care.

As mentioned above, we had the opportunity to access and analyze documents issued by the organization, including an evaluation report with data related to the results delivered by the systems produced at the end of 2010 for the Program Board. In this report the Director of eHealth pointed to some of the economic outcomes produced by the EMR system representing the non-human super actor:

*“We did some examinations and we had a conservative estimate of how much time it saves in the departments by having details electronically and we found that they saved hundreds of Mondays a year. We estimate that by stopping printing all clinical discharge letters for GPs, we will be printing 1 million less pieces of paper a year. And then you have to put each one of them in an envelope and send them to GPs. This means that there is potential for savings from an administrative perspective and it can help make us more efficient.” (Director of eHealth, extracted from evaluation document).*

According to this document, adopting the system improved the delivery of care, offering clinicians immediate access to results. It also eliminated the need for repeated tests due to lost reports and improved laboratories’ ability to handle and respond to test requests. Five years after the adoption of the system, it was also acknowledged that it had improved the clinical information provided electronically to laboratories. Data on the percentage of blood tests ordered electronically in July 2010 is worth noting: 99 % of blood tests were ordered electronically by the A&E ward, 97 % of blood tests by in-patient wards and 89 % by outpatient wards. The same results were found for the ordering of x-rays in Radiology. The possibility to send electronic GP referrals helped save more than 300 h per month. Communications with clinical wards took 1 day less compared to before the adoption. Electronic discharge letters also helped save time for communications with GPs, since letters are now received 3 days earlier than before EMR adoption.

This is a selection of the information provided in the report, offering an idea of the indicators used for monitoring system performance and the results achieved in previous years.

We carried out observations on various wards including A&E, one of the busiest in the hospital, taking account of over 100 hundred patient admissions. We observed a very organized reception and efficient admission of patients: when patients arrive at reception, the receptionist asks the patient or his/her attendant if this is their first admission. If the patient has been already admitted, all his/her information is already stored in the EMR; if not, some basic information has to be provided. The triage nurse assesses the patient’s condition at the time of arrival at A&E, identifies the problem and allocates a triage classification. This information is entered into the EMR system, which is also used to book a bed on the ward and check all the relevant information.

During the time we spent on the ward, some patients were treated and discharged. In this case, the receptionist accessed the patient’s EMR and sent the discharge letter to the GP electronically. In other cases, patients were sent to a different ward after being seen in A&E and a bed was booked in the new ward via the EMR system.

Based on the observations we carried out, some actors showed great confidence in using the system while others appeared to be less experienced.

*The doctors had tablet PCs to use during their daily rounds. In addition there were several computers and printers located in spaces within each ward. Clinicians and nurses were also observed to be using laptops during their field visits. This use of technology appears to have influenced patients’ awareness that the system was being used within the hospital and their perception that the organization seemed to be ‘integrated’. Furthermore, when patients move around the hospital they are not asked several times for the same information. This seems to have produced a ‘safer feeling’ in patients: they know all their data is stored together and easily accessible and this gives them a good impression and helps them feel better. (Researcher’s field notes).*

We also observed that many tools were used in the process: the EMR system, clinical records, diaries, sheets of paper, and post-it notes, however the most important tool appeared to be the computer and the EMR system. These objects play a part in the admission process and comprise ‘ordered relations that materialize in patient/nurse interaction’ (as affirmed by Bruni [55]). All these objects are closely connected and all the information they contain is subsequently integrated in the EMR system representing the non-human super actor. However, these non-human objects require human intervention, even if they guide human interaction and involve other objects.

## Discussion

Drawing on the theoretical perspective of Actor Network Theory, this study set out to examine the relationships between human and non-human (ICT) actors in the implementation of a complex sociotechnical change represented by the implementation of an EMR system by a large teaching hospital. It used a mixed-methods case-study approach, involving key respondent interviews, observations and document analysis, in order to gain a rich picture of the context of the implementation. The results illustrate how people can mediate the goals, adoption and impacts of information systems during their design, implementation and use, thus supporting the corpus of knowledge on the importance of human and organizational factors for achieving successful systems adoption [25–27].

From a theoretical perspective, they demonstrate how a major IT initiative, embodied here by the EMR system, can act as a focal point for the achievement of multiple organizational goals and for reconciling the information requirements of different stakeholders, as well as the importance of user-centered design, perceived and experienced benefits, and strategic leadership, for encouraging change and impact. Taken together, these findings support the concept of the EMR system as a ‘non-human super actor’ around which the efforts of other actors in the system coalesce and whose success is mediated by the ways in which these other actors interact with, respond to and derive utility from it, as well as the network and whole system effects such complex interventions can bring.

The interviews and observations also helped to shed light on the impacts on the people working within the organization, on patients and on relationships with stakeholders within and outside of the hospital [12]. A set of indicators was defined for monitoring impacts in terms of efficiency and savings, and revealed that the adoption of the system produced real and measurable benefits and impacts on hospital performance.

The adoption of the system produced visible goals defined at the initial phase and over the following 5-year period, helping to show the efficiency of the system and its effectiveness for staff. Continuous adjustments by the Department of eHealth were based on the results of routine reviews and meetings with key actors working on the wards. In conclusion, the case study revealed the importance of obtaining the strong commitment of all the actors involved in the EMR network, supported by an expectation of appreciable results in the long term.

This included stakeholder participation in the adoption, implementation and evaluation process, including inviting users to take part in the introduction of the system from the very beginning, and requiring them to understand why change was necessary, so that the concept of envisioning an immediate gain as argued by Callon [17] then becomes important.

The emergence of the EMR network is shown in the traces of actions and activities revealed in the documents and in the accounts of the actors interviewed. The key role of the Director of eHealth and his team were identified by several interviewees. He made the definition and adoption of a new system recognizable for others, made its acceptance an obligatory passage point for entering the network, and he became indispensable in the process, playing a focal role.

Furthermore, as the interviews revealed, clinicians generally accepted changes once they saw that these facilitated their workload and/or improved the quality of their work. This indicates interessement [17]. During this phase, each entity (i.e. clinicians, nurses, other member of staff, as well as documents, and existing technologies) was invested by some interest in adopting the new EMR system. In this way, a pattern of exchange emerges establishing what each of the entities will get in return for accepting to be involved in the network. This point also illustrates the importance of not focusing solely on the ability of the EMR system to support individual users conducting single tasks, but of emphasizing the potential effects of the EMR system as a network on clinical workflow.

A department of eHealth was set up at the hospital staffed by computer-literate people, including technicians as well as employees who previously worked on the wards. They recognize information is a key organizational resource that is central to all its functions. The introduction and implementation of IT is fully integrated into the process of organizational change and is driven by project objectives. In this process, the non-human actor – the EMR system - dominates everyday activities within the hospital. With the enrolment, in fact, the entities in the emerging network were coordinated and aligned: new roles and activities were identified to support the new system.

Furthermore, as discussed before, a crucial step in this phase was the identification of “super users” in each ward. These are people (clinicians and nurses) capable of training other people. This emphasizes that user-involvement is important to foster the ownership of the systems that will actually match work processes. It is not enough to include a few potential users in the project group and have them negotiate the system specifications, discuss implementation plans and the achievement of organization change in a meeting.

Social-technical approaches favor a central role of the users throughout the development process, even if defining how to involve these users is not easy [56]. In fact, it often happens that users are only consulted a few times in meetings whose setup mitigates any real involvement of users or any real openness of the designers [57].

The adoption of particularly innovative and complex information systems requires adequate planning for their implementation, which must involve the identification of the barriers and facilitators to EMR adoption and implementation that are perceived by different groups [58] and the definition of an impact measuring method to assess the effects of decisions made by actors in the organization and in order to guide future ones [59].

Consensus is also growing with regard to the role of EMRs in the hospital due to the evidence of its efficiency and effectiveness during recent years. It is possible that eHealth will have the capability to change the clinical relationship with patients. The mobilization phase showed that the network starts to speak as one and to operate as a recognizable ‘actor’ at the end, producing great effects within the hospital.

EMRs have the potential to empower patients by offering greater access to their personal data, health care information, and communication tools, which may aid self-care, shared decision-making and clinical outcomes. As macro or super actors these systems have the potential to shape the behaviors of a wider range of actors. However, there are many challenges, including the need for ongoing user involvement and an evaluation process that must be mobilized to produce the transformative vision of the ICT proponents.

Implementing change affecting an organization’s structure, culture, work processes, behavior and communication channels can be considered one of the most difficult and challenging tasks when carrying out an innovation project at a healthcare organization. The facilitation of networks, such as the EMR network, to promote active “change management” at all system levels is likely to encourage better implementation of EMR systems.

We decided to use ANT based on the fact that previous studies have demonstrated that the approaches to ICT implementation used in other industries had limited success in the health care sector [60] The limitation of these traditional approaches to ICT work practice is evidenced in the large number of reported failures of large health IT projects [5]. This has led to a search for approaches that place greater emphasis on the interconnectedness between the social (people, values, norms, culture) and technical (tools, hardware, equipment, processes) aspects of organizations and thus we decided to consider documents, proceedings and ICT tools as key players within this innovation process. As a result, we considered ANT as an analytical technique where the researcher follows actors and tries to understand what they do. It represents a valuable method for understanding and recognizing the value of complex realities, which may be neglected by more positivistic and cause-effective approaches [61, 62].

Using the mixed methods of documentary analysis, interviews and observations for the case study was useful for converging different types of enquiry. The interviews helped to shed light on the process of actor-network formation and the centrality of the EMR system as a central non-human super actor, and confirmed the importance of key barriers and facilitators for changing management as already reported in existing health IT literature. The written documents provided useful benchmarks for progress during the project and information on the wider context of organizational decision making at the time. Undertaking live observations of the system in use allowed us some interesting insights into workflow issues that the system had addressed.

The study was not designed to observe the process of implementation from start to finish and relies on hindsight observations of interviewees to some extent. However there are sound reasons for using a retrospective case study approach in order to examine a successfully integrated EMR system, since this provides a rapid, yet fairly comprehensive means of identifying the various factors that were critical in its adoption, which can sometimes take several years to complete in full.

## Conclusions

Previous analyses of EMR implementation have tended to focus more on the systems themselves than on the sociotechnical aspects of the implementation processes, although literature is evolving on this topic [4, 10, 12–14, 63]. This study adds to this literature by examining how using Actor Network Theory as an interpretive framework can help to shed light on the various social processes through which systems come to be accepted, adapted and adopted. The results indicate that it is legitimate to interpret the system as a strong non-human super actor within a heterogeneous network of actors, and to describe the processes of stakeholder engagement, alignment and activation with reference to the phases of problematization, interessement, enrolment and mobilization. This may be helpful for managers seeking to implement such programs by indicating what to expect at the different stages, how to recognize the role of the system as part of an interacting social network and how to leverage these insights to help align the goals and actions of the stakeholders around the technology, for more rapid adoption and more effective impact.

The results of this study are consistent with ANT in illustrating the important role of the IT system as a central super-actor, around whom the various other actors in the network became aligned, seeing the success of the project as a shared endeavor serving mutually valuable goals for which engagement and action were warranted. It also highlights the importance of user-involvement in fostering ownership of the new systems by ensuring that they support or enhance existing work processes, rather than simply adding further complexity.

Many countries have defined national policies and guidelines for the introduction of EMR systems and many hospitals are either engaged in, or planning their implementation. Our findings offer several suggestions on how to achieve a smoother transition. The process of implementation should be informed by an understanding of its micro and macro-contexts, taking into account stakeholder needs at organizational level and policy goals and objectives in more general terms. EMR are complex and multifaceted systems serving different stakeholders in different ways and implementing EMR should be conceived of as much as a change management exercise as an IT program. Strong, long-term commitment is needed in order to manage the changes in roles and workflow introduced by the EMR system, including efforts to generate a positive culture of change through a clear training program delivered by people familiar with the clinical tasks and issues (super users), defining any new roles that may be required within the healthcare setting and realigning the organization’s structure, if appropriate. Since effective EMR implementation is a lengthy process that evolves over time, it is also important to engage in on-going assessment in order to investigate the nature and direction of impacts and put prompt and well-timed actions in place to address gaps and barriers.

Based on our study we recommend that managers responsible for EMR implementation consider using ANT to help guide the planning and monitoring of change processes, in ways that will facilitate effective system adoption for the benefit of the organization and its various stakeholders.

## Abbreviations

ANT: Actor Network Theory
EMR: Electronic Medical Record
ICT: Information and Communications Technology
